# The Potential of Biomaterial-Based Approaches as Therapies for Ischemic Stroke: A Systematic Review and Meta-Analysis of Pre-clinical Studies

**DOI:** 10.3389/fneur.2019.00924

**Published:** 2019-08-27

**Authors:** Faye Bolan, Irene Louca, Calvin Heal, Catriona J. Cunningham

**Affiliations:** ^1^Division of Neuroscience and Experimental Psychology, Faculty of Biology, Medicine and Health, School of Biological Sciences, University of Manchester, Manchester, United Kingdom; ^2^Faculty of Biology, Medicine and Health, Centre for Biostatistics, Academic Health Sciences Centre, University of Manchester, Manchester, United Kingdom

**Keywords:** systematic review, meta-analysis, stroke, biomaterials, tissue engineering, hydrogels, nanoparticles, regenerative medicine

## Abstract

**Background:** In recent years pre-clinical stroke research has shown increased interest in the development of biomaterial-based therapies to promote tissue repair and functional recovery. Such strategies utilize biomaterials as structural support for tissue regeneration or as delivery vehicles for therapeutic agents. While a range of biomaterials have been tested in stroke models, currently no overview is available for evaluating the benefit of these approaches. We therefore performed a systematic review and meta-analysis of studies investigating the use of biomaterials for the treatment of stroke in experimental animal models.

**Methods:** Studies were identified by searching electronic databases (PubMed, Web of Science) and reference lists of relevant review articles. Studies reporting lesion volume and/or neurological score were included. Standardized mean difference (SMD) and 95% confidence intervals were calculated using DerSimonian and Laird random effects. Study quality and risk of bias was assessed using the CAMARADES checklist. Publication bias was visualized by funnel plots followed by trim and fill analysis of missing publications.

**Results:** A total of 66 publications were included in the systematic review, of which 44 (86 comparisons) were assessed in the meta-analysis. Overall, biomaterial-based interventions improved both lesion volume (SMD: −2.98, 95% CI: −3.48, −2.48) and neurological score (SMD: −2.3, 95% CI: −2.85, −1.76). The median score on the CAMARADES checklist was 5.5/10 (IQR 4.25-6). Funnel plots of lesion volume and neurological score data revealed pronounced asymmetry and publication bias. Additionally, trim and fill analysis estimated 19 “missing” studies for the lesion volume outcome adjusting the effect size to −1.91 (95% CI: −2.44, −1.38).

**Conclusions:** Biomaterials including scaffolds and particles exerted a positive effect on histological and neurological outcomes in pre-clinical stroke models. However, heterogeneity in the field, publication bias and study quality scores which may be another source of bias call for standardization of outcome measures and improved study reporting.

## Introduction

Stroke is a global health problem with limited treatment options. The World Health Organization reports stroke as the second leading cause of death worldwide accounting for around 6.7 million deaths annually (1). For patients surviving stroke, close to two thirds will have a disability (2). Despite the major societal impact, the only widely available therapy for ischaemic stroke is tissue plasminogen activator (tPA). However, due to the narrow time window of administration (<4.5 h of symptom onset), only around 12% of patients in the UK are eligible for treatment (3). In recent years, great advances have been made in the development of endovascular thrombectomy as an alternative treatment (4, 5). While clinical trials have demonstrated thrombectomy is effective up to 6 h after stroke onset, it has been estimated that only 10% of patients would be eligible even with national coverage in the UK, which is presently not the case (6). Both thrombolysis and thrombectomy aim to restore reperfusion and limit further damage to the ischaemic brain and there are currently no approved regenerative medicine therapies available for promoting repair and regeneration.

In recent years, interest in regenerative medicine approaches to increase neuronal tissue repair following ischaemic stroke has increased. Notably, over 20 early phase clinical trials have been conducted assessing the safety of cell therapies including mesenchymal stem cells (MSCs) (7), neural stem cells (NSCs) (8), and haematopoietic stem cells (9). A number of studies have used cell-based therapies and growth factors to promote endogenous brain repair or tissue replacement in animal models of stroke with limited success. For this reason, biomaterials are now being investigated as potential agents to enhance the therapeutic efficacy of such interventions as scaffolds for tissue regeneration or vehicles for drug release (10, 11). For example, cell transplantation in the brain may be facilitated by the use of engineered scaffolds, which mimic native extracellular matrix (ECM) properties and provide adhesion sites for native cell attachment, aiding graft cell retention in the infarct cavity (12, 13). Alternatively, scaffolds may be used to aid recruitment and survival of endogenous stem cell populations for tissue restoration. Examples of scaffolds studied for cell delivery in stroke models include natural and synthetic hydrogels, electrospun fibers, sponges, and glue. Particulates are another class of biomaterials commonly used as delivery systems for targeted and controlled release of therapeutic agents. A wide variety of particulates have been tested for ischaemic stroke repair including nanoparticles, microparticles, micelles, liposomes, nanocarriers, and microspheres (14, 15).

To date, there is no systematic review or meta-analysis available investigating the potential of biomaterial-based approaches in pre-clinical models of ischaemic stroke. Given that biomaterial-based interventions are currently attracting great interest it is timely therefore to provide such a review, with the hope of averting the “translational roadblock” (16) and poor predictive validity of previous studies of neuroprotective agents for stroke (17, 18). Our systematic review and meta-analysis aims to assess the methodological quality of current research into biomaterial-based interventions for ischaemic stroke repair and to determine the efficacy of such approaches, helping to inform future research directions.

## Materials and Methods

### Search Strategy

We searched PubMed and Web of Science for (stroke OR cerebral ischemia OR cerebral ischemia) AND (biomaterial OR tissue engineering OR hydrogel$ OR scaffold$ OR ^*^particle$ OR sponge$). No restrictions were placed on date of publication and the last search was conducted on 4 February 2019. We also searched reference lists of review articles for additional articles. Titles and abstracts were initially screened by two authors (CC and FB).

### Data Sources, Studies Selections, and Data Extraction

Full texts of the identified publications were screened (CC, FB, and IL) for studies assessing the efficacy of biomaterial-based strategies in pre-clinical models of cerebral ischemia. Studies were included if lesion volume and/or neurological score were reported as outcome measures. Studies in which the method of induction of cerebral ischemia was not stated were excluded. After screening full texts for suitability, study design information was extracted from each publication (CC and FB). Information extracted included the species and stroke model used, type of biomaterial investigated and whether the material was combined with another therapeutic agent (such as growth factors or a drug), the time of administration and any functional tests conducted.

The risk of bias of each publication was assessed using the CAMARADES (Collaborative Approach to Meta-analysis and Review of Animal Data in Experimental Studies) study quality checklist (19): (1) peer reviewed publication; (2) control of temperature; (3) random allocation to treatment or control; (4) blinded induction of ischemia; (5) blinded assessment of outcome; (6) use of anesthetic without significant intrinsic neuroprotective activity; (7) animal model (aged, diabetic, or hypertensive); (8) sample size calculation; (9) compliance with animal welfare regulations; and (10) statement of potential conflict of interests. All information extracted was independently cross-checked by a second reviewer (CC, FB, and IL).

For both lesion volume and neurological score, mean values, ± standard deviation (SD) or standard error of the mean (SEM), and group sizes for treatment and control groups were extracted. Publications that did not specify specific group sizes and reported variance as SEM or did not include control groups were excluded from the meta-analysis. Where variance was reported as SEM, this was converted to SD in Microsoft Excel in order to calculate standardized mean difference (SMD) for analysis. Since the effect of biomaterial administration on stroke recovery was the main focus of this analysis, control groups chosen for comparisons were either stroke only or vehicle. In cases where the material was combined with additional therapeutic agents, stroke or vehicle were also used as controls. In two cases (20, 21), the control group was biomaterial alone so these papers were excluded from the meta-analysis.

Two publications which presented lesion volumes and neurological scores as median ± interquartile range (IQR), were also excluded from the meta-analysis (22, 23). One paper expressed the neurological score inversely to the convention (higher scores indicate a higher degree of impairment) so the data were inverted relative to the stated baseline measure (24). Where multiple treatments were assessed, we selected the treatment identified by authors in the publications as the primary focus. When publications reported multiple outcome measures from multiple timepoints, the data for the final end point was chosen. Where publications included multiple administration timepoints, data from each group was extracted separately.

Where possible, raw values were extracted from the text of the publication. When data was presented only graphically, the online tool WebPlotDigitizer (https://automeris.io/WebPlotDigitizer/) was used to estimate mean and variance from graphs. Estimate measures were independently cross-checked by a second reviewer and any conflicts (>10% difference) were resolved by a third reviewer (CC, FB, and IL). For analysis of effect size, the publications were split into subgroups by type of biomaterial (scaffolds and particles). The scaffolds subgroup included publications using hydrogels, extracellular matrix scaffolds, fibrin glue, and sponges. The particles subgroup included publications using nanoparticles, microparticles, nanocarriers, microspheres, liposomes, nanoemulsions, nanotubes, and micelles. The selected publications were also divided into subgroups according to the time of administration. The groups were as follows: administration prior to stroke and up to time of reperfusion; from reperfusion to 24 h post-stroke; >24 h to 1 week post-stroke; >1–3 weeks post-stroke and multiple administrations spanning these timepoints.

### Statistical Analysis

For both the lesion volume and neurological score outcomes, the SMD between the trial arms and accompanying 95% confidence intervals were calculated using DerSimonian and Laird random effects meta-analysis. Studies were weighted based on animal number. The presence of heterogeneity in the data sets was assessed using the *I*^2^ statistic. Due to very high heterogeneity in the data (*I*^2^ = 82.8 and 84.3% for lesion volume and neurological score, respectively), random effects were chosen. Forest plots were used to visually present the results. The analysis was stratified by type of biomaterial and time of administration although for brevity, only the former are presented graphically. The extent of publication bias was assessed graphically using funnel plots and confirmed with Egger's regression test. The trim and fill approach was also used to estimate an effect size accounting for publication bias. Stata 15 (StataCorp, USA) was used for all statistical analyses with the exception of the trim and fill analysis which was conducted in RStudio version 1.1.463 (RStudio Inc., USA) using the metafor package (http://www.metafor-project.org/doku.php).

## Results

### Study Selection and Characteristics

**Figure 1:**
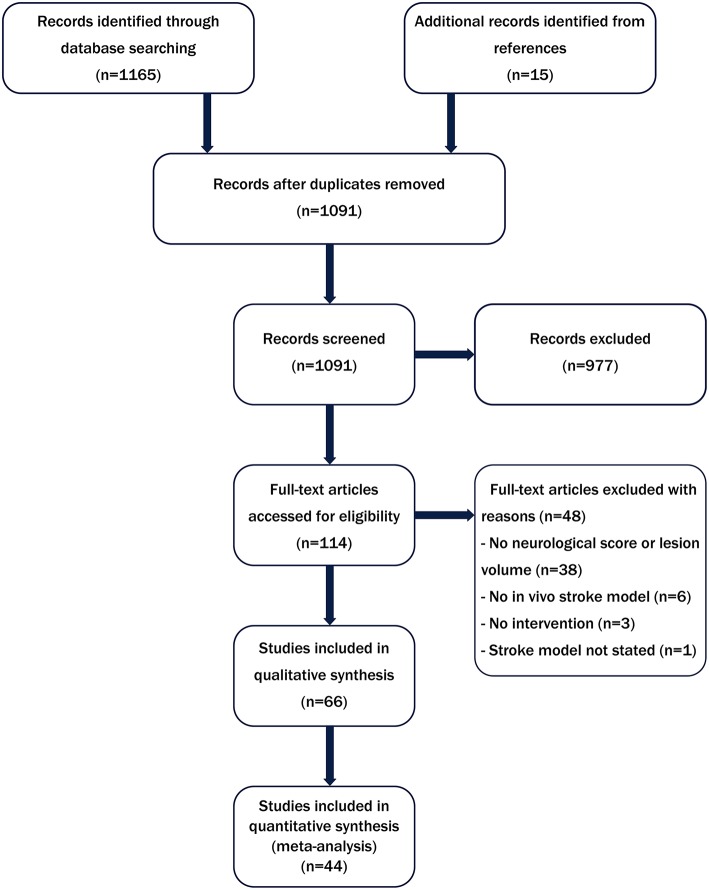
Flow diagram summarizing the literature search strategy and number of studies included in the systematic review and meta-analysis.

As shown in Figure 1, 1,091 publications were identified from our literature search of which 66 met the inclusion criteria for the systematic review. The characteristics of these studies including stroke model, animal numbers and type of biomaterial intervention are shown in Supplementary Table 1. All of the included studies were conducted in rats (*n* = 49) or mice (*n* = 16) or both rats and mice (*n* = 1). The most commonly used model was the transient intraluminal filament model of middle cerebral artery occlusion (MCAO) (*n* = 46). Only two studies used comorbid animals. Hayon et al. (25) used spontaneously hypertensive rats and Fabian et al. (26) induced acute hyperglycemia in Sprague-Dawley rats using streptozotocin. As shown in Supplementary Table 1, there was high variation in the functional outcome measures employed (23 different behavioral tests). Furthermore, several different neurological scores were reported including modified neurological severity score (both 14 and 18 points), Bederson, modified Bederson and Longa (27, 28). A total of 44 studies including 1,075 animals (control *n* = 522, treatment *n* = 553) were then included in the meta-analysis reporting 86 comparisons. Of these, 51 assessed lesion volume and 35 assessed neurological score. There was substantial heterogeneity in the datasets from both lesion volume and neurological score outcomes (*I*^2^ = 82.8 and 84.3%, respectively).

### Synthesized Findings

**Figure 2:**
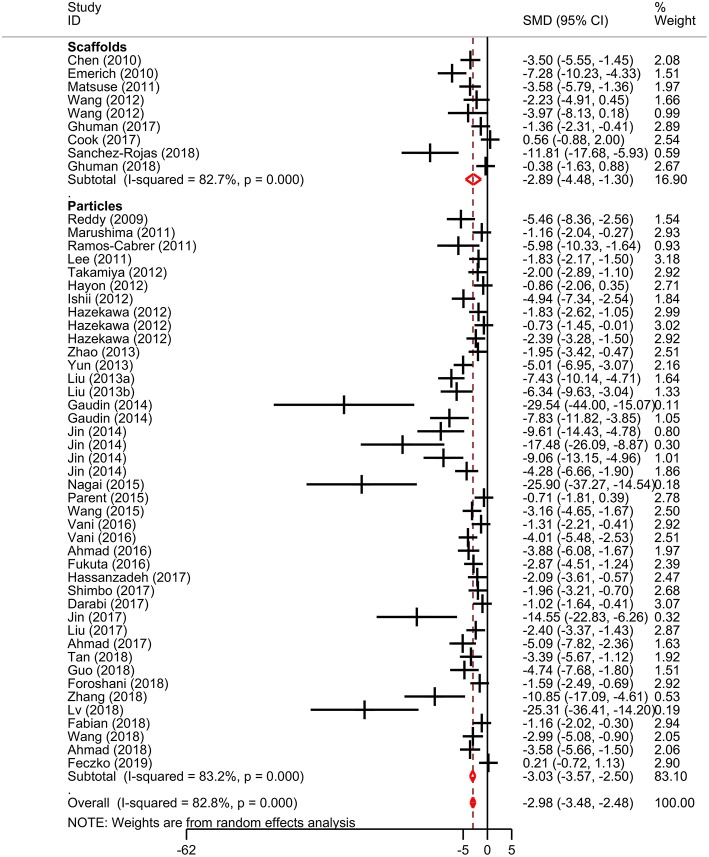
Effect sizes for biomaterial-based interventions for lesion volume. Forest plot of standardized mean difference and 95% CI. CI, confidence interval; SMD, standardized mean difference.

**Figure 3:**
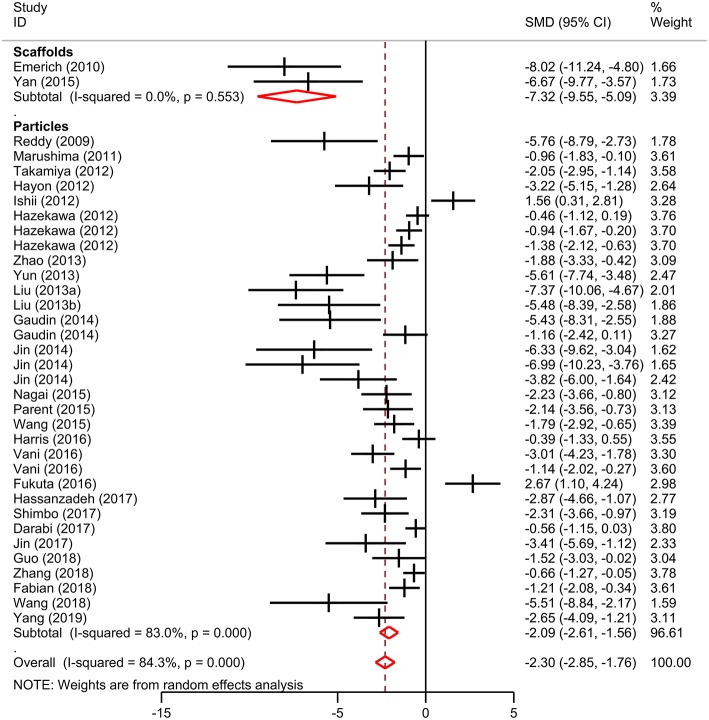
Effect size for biomaterial-based interventions for neurological score. Forest plot of mean standard difference and 95% CI. CI, confidence interval; SMD, standardized mean difference.

Meta-analysis was performed on lesion volume and neurological score data. Overall, treatment with biomaterial-based interventions led to improvements in lesion volume (SMD: −2.98, 95% CI: −3.48, −2.48). Both the scaffolds (SMD: −2.89, 95% CI: −4.48, −1.30) and particles subgroups (SMD: −3.03, 95% CI: −3.57, −2.50) had comparable effect sizes (Figure 2). There was high variability in the reported effect sizes in the particles groups ranging from **–**29.54 to −0.21. Similarly, biomaterial-based approaches were also associated with overall improvements in neurological score (SMD: −2.3, 95% CI: −2.85, −1.76; Figure 3). Administration of the biomaterial-based therapies within 24 h of stroke onset appeared to be most effective leading to the most marked improvement in lesion volume (SMD: −3.94, 95% CI: −5.19, −2.69; Table 1).

**Table 1:** Subgroup meta-analysis comparing time of intervention on lesion volume and neurological score outcomes.

	**Lesion volume**			**Neurological score**		
**Time of intervention**	**Comparisons**	**Effect size (95% CI)**	***I^**2**^, p-*value**	**Comparisons**	**Effect size (95% CI)**	***I^**2**^, p-*value**
Pre-treatment−0 h	25	−3.15 (−3.76, −2.55)	79.7%, <0.001	18	−3.03 (−4.10, −1.96)	89.2%, <0.001
>0–24 h	15	−3.94 (−5.19, −2.69)	87.1%, <0.001	13	−1.84 (−2.48, −1.21)	75.3%, <0.001
>24 h−1 week	5	−2.15 (−4.43, −0.14)	85.5%, 0 < 001	0	N/A	N/A
>1–3 weeks	3	−1.14 (−2.27, −0.00)	41.1%, 0.183	0	N/A	N/A
Multiple timepoints	3	−3.51 (−6.75, 0.27)	78.5%, 0.010	4	−1.32 (−2.18, −0.45)	56.5%, 0.075

### Risk of Bias and Study Quality

Risk of bias was assessed using the CAMARADES checklist (19). Overall, the median score was 5.5/10 (IQR 4.25-6). Studies within the scaffolds (6/10, IQR 4-7) and particles (5/10, IQR 4-6) subgroups had very similar scores (Table 2). Reporting of randomization (56%), blinding (35% to stroke and 50% to outcome) and sample size calculations (9%) was low.

**Table 2:** Summary of study quality assessed by study compliance to the CAMARADES risk of bias checklist.

	**Overall**	**Scaffolds**	**Particles**
(1) Peer-reviewed publication (%)	100	100	100
(2) Control of temperature (%)	50	42.1	53.2
(3) Random allocation to treatment or control (%)	56.1	52.6	57.4
(4) Blinded induction of ischemia (%)	34.9	42.1	31.9
(5) Blinded assessment of outcome (%)	50	52.6	48.9
(6) Use of anesthetic without significant intrinsic neuroprotective activity (%)	83.3	89.5	80.9
(7) Animal model (aged, diabetic, or hypertensive) (%)	3	0	4.26
(8) Sample size calculation (%)	9.1	15.8	6.4
(9) Compliance with animal welfare regulations (%)	93.9	89.5	95.7
(10) Statement of potential conflict of interests (%)	62.1	73.7	57.4
Median quality (/10) (IQR)	5.5 (4.25–6)	6 (4–7)	5 (4–6)

**Figure 4:**
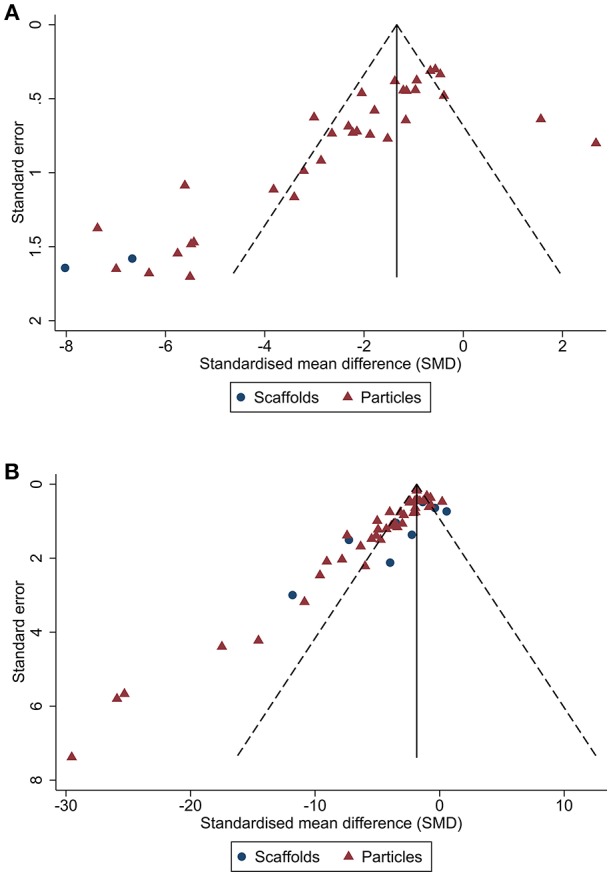
Publication bias analysis for biomaterial-based interventions by type. Funnel plots with pseudo 95% CI for publication bias of lesion volume **(A)** and neurological score **(B)**.

**Figure 5:**
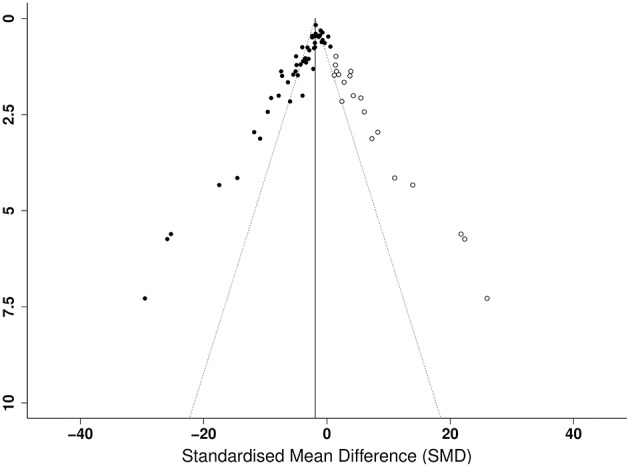
Trim and fill analysis of lesion volume showing published studies (filled circles) and estimated unpublished studies (unfilled circles). The solid vertical line indicates the adjusted effect size.

Publication bias was then assessed. From visual inspection of the funnel plot for lesion volume (Figure 4), there was pronounced asymmetry denoting publication bias. This was confirmed by Egger's regression test (*p* < 0.001). Similarly, there was asymmetry in the funnel plot of neurological score (*p* < 0.001; Figure 4). Trim and fill analysis estimated 19 “missing” studies on the right side of the funnel plot of lesion volume outcome (Figure 5) which adjusted the effect size to −1.91 (95% CI: −2.44, −1.38). Conversely, trim and fill analysis did not report any “missing” studies for neurological score.

## Discussion

### Summary of Findings

In this study we assessed the efficacy of biomaterial-based approaches in pre-clinical models of ischaemic stroke. A total of 66 publications met our inclusion criteria for the systematic review with 44 studies (86 comparisons) included in the meta-analysis. Overall, our findings show that biomaterial-based interventions improved both lesion volume and neurological score. Treatment within 24 h of stroke onset appeared to be the most effective timepoint. We next assessed risk of bias using the CAMARADES checklist (19) and showed the median score was 5.5/10. We also identified pronounced asymmetry in the funnel plots of both the lesion volume and neurological score dataset, indicating publication bias. Trim and fill analysis indicated there were 19 “missing” studies reporting negative or neutral lesion volume data which when adjusted for, greatly reduced the effect size.

### Variability in Outcome Measures

In agreement with a recent systematic review on animal models of stroke and vascular cognitive impairment (29), we reported very high variability in the choice of functional outcome measures. A limitation of the pre-clinical stroke field is the lack of consensus on the optimal test or battery of tests for assessing functional recovery (30). We selected neurological score as one of our outcome measures as it is by far the most widely used assessment of recovery (29). While there are a number of advantages including being fast to perform and requiring no specialist equipment, a large drawback of neurological scores is that they are highly subjective (31). Additionally, we noted there was no standardization in the scale used and a number of publications neglected to report the scoring system used. A further limitation is that as rodents have a great capacity for spontaneous recovery, simpler assessments such as neurological scores often cannot detect deficits at later timepoints (30). These scores may therefore have limited use in studies where scaffold materials are administered at chronic timepoints after stroke (1–2 weeks) (32–34). In addition, neurological scores are not able to adequately distinguish compensatory strategies from true motor recovery (35) which as previously suggested, could lead to false positives (36, 37). Future studies should therefore aim to use tasks such as skilled reaching and gait analysis which can differentiate between the two.

Additionally, we noted that all but two publications identified in our systematic review reported neurological score as mean ± SD/SEM. This is surprising given that these data are ordinal and thus should be expressed as median ± IQR. The calculated effect sizes of individual studies should therefore be approached with caution. Nevertheless, given that the central limit theory states that the mean of a large number of observations will tend toward a normal distribution, our overall effect size for neurological score are robust.

Lesion volume was also chosen as an outcome measure in our meta-analysis given its wide use in the pre-clinical stroke field. The vast majority of the included studies assessing the efficacy of nanoparticles chose acute end points (24–48 h) with lesion volume as the primary outcome measure of neuroprotection. However, most studies assessing the capacity of scaffolds to promote brain regeneration elect to administer during the sub-acute and chronic phase of stroke. In these instances, it is currently unclear whether lesion volume assessment at chronic timepoints is a measure of tissue atrophy, repair or a combination of the two (33, 38, 39). Future work should therefore focus on developing standardized methods for evaluating tissue regeneration. For example, Ghuman and colleagues have chosen to focus on host cell infiltration into ECM hydrogels as an outcome measure (32, 38). Furthermore, it is worth noting that many studies that focused solely on material characterization or assessment of administration at the tissue level were excluded from our systematic review and meta-analysis as lesion volume and neurological scores were not included as outcome measures.

Both histological techniques (including 2,3,5-triphenyltetrazolium chloride (TTC), cresyl violet or haematoxylin & eosin (H&E) stains) and magnetic resonance imaging (MRI) were used to measure lesion volume. The variation in choice of method likely reflects the availability of MR scanners or preference of researchers toward a certain method. However, this may introduce inter-study variability into lesion volume measurements. A meta-analysis comparing measurements obtained from T2-weighted MR images and histological sections found a strong correlation between the two methods (*p* < 0.001) (40). Nevertheless, lesion volume measurements from MR images were larger than for histology and there was considerable variation in the reporting and use of MR methods (40). Despite this, as our study compares the SMD of treatment and control group measurements obtained using the same method, the differences in methodology is unlikely to have an effect on the conclusions of our study.

### Study Quality and Risk of Bias

To improve the quality of pre-clinical research, a number of guidelines have been published in recent years including the National Centre for the Replacement, Refinement and Reduction of Animals (NC3Rs) published the Animal Research: Reporting of *in vivo* Experiments (ARRIVE) guidelines (41). This consists of a checklist of 20 items which should be reported including full details of allocation to experimental groups, sample size calculations and reporting of exact animal numbers. While most of the studies included in our systematic review were published after the ARRIVE guidelines (2011 onwards), reporting of these items remained low. This is in agreement with a recent position paper which shows compliance with the guidelines so far has been low (42). We noted that several publications had extremely large effect sizes (SMD >-10) of which a number reported >80% reductions in lesion volume following treatment. Previous systematic reviews of *in vivo* studies have shown that studies reporting the greatest efficacies often have the lowest compliance with such checklists (43). It is possible that limitations in study design could account for an overestimation in effect size and reduced reliability of the results. It has been suggested that the lack of blinding and randomization has led to false positives in neuroprotection studies or over-estimates of efficacy which may have contributed to the translational roadblock in the field (16).

As previously mentioned, our results indicated the presence of publication bias. In particular, trim and fill analysis predicted there was a substantial number of “missing” unpublished studies reporting neutral or negative lesion volume outcomes. Publication bias is an issue not limited to pre-clinical stroke research (19, 44, 45) which can lead to overestimation of efficacy. For example, a number of studies has shown that conference abstracts reporting positive results are more likely to be published later (46–48). To avoid another translational roadblock, greater emphasis should be placed on publishing negative and neutral data.

### Limitations

This systematic review and meta-analysis is the first to collate the pre-clinical literature on biomaterials for ischaemic stroke. As such, we believe the synthesized findings and conclusions are important for the field. Nevertheless, there are a number of limitations. Firstly, only summary data obtained from the publications was used for the meta-analysis and in many cases, values had to be estimated from graphs. As such, our measurements may differ marginally to the raw values. However, we did not contact authors to obtain these data. A study by the Cochrane Library compared meta-analyses using summary or “aggregate” data to those using raw data and found the difference in results and conclusions to be minimal (49). As our aim was to provide a succinct summary of the current field, we reason that the use of summary data for meta-analysis has negligible impact on the conclusions of the review.

Additionally, the considerable heterogeneity in the types of biomaterials used made identifying suitable subgroups problematic. We reasoned that all of the publications could be classified broadly as either scaffolds (administered at the lesion site for *in situ* tissue engineering in the sub-acute to chronic phase of stroke) or as particles (usually delivered systemically before or acutely after stroke). We acknowledge the wide range of material types within the chosen subgroups and an alternative option may have been to include a greater number of specific subgroups. However, we reasoned that this would decrease the statistical power of the meta-analysis and the main objective was to give an overview of the efficacy of biomaterial-based approaches in pre-clinical stroke.

We acknowledge our search criteria only identified studies relating to ischaemic stroke. A separate search containing the keywords intracerebral hemorrhage and subarachnoid hemorrhage, returned seven relevant publications (50–56). Based on the small number of publications and the heterogeneity in outcome measures used, we chose not to include these studies. Given our findings concerning risk of bias and study quality, a future systematic review and meta-analysis should investigate this in the emerging field of biomaterial therapies for hemorrhagic stroke.

## Conclusions

Our meta-analysis revealed that studies assessing biomaterial-based interventions for ischaemic stroke report overall positive results leading to reductions in lesion volume and neurological improvements in rodent models. Additionally, our systematic review regarding study quality showed CAMARADES checklist score was 5.5/10. Our findings provide insight into the field of biomaterials for stroke therapeutics and the quality of studies conducted. We believe the results highlight the need for improved study design and reporting to ultimately support translation of biomaterial-based therapies to the clinical setting.

## Data Availability

All datasets generated for this study are included in the manuscript and/or the Supplementary Files.

## Author Contributions

CC: conception. CC, FB, and IL: design, literature search, study screening, data extraction, and manuscript writing. CH and CC: data analysis. CC, CH, FB, and IL: interpretation.

### Conflict of Interest Statement

The authors declare that the research was conducted in the absence of any commercial or financial relationships that could be construed as a potential conflict of interest.
