# Supplementary material for: The Potential of Biomaterial-Based Approaches as Therapies for Ischemic Stroke: A Systematic Review and Meta-Analysis of Pre-clinical Studies
Source: Front Neurol. 2019 Aug 27;10:924. doi: 10.3389/fneur.2019.00924 (PMC6718570; doi:10.3389/fneur.2019.00924)
Supplement: Supplementary file 1 [file Table_1.docx]

#### Table S1

Summary of all 66 studies included in the systematic review.

| **First Author** | **Year** | **Species** | **Animals** | **Stroke Model** | **Biomaterial** | **Combined with** | **Treatment Timepoint(s)** | **Route** | **Lesion**  **Volume** | **Functional Assessments** |
| --- | --- | --- | --- | --- | --- | --- | --- | --- | --- | --- |
| Ahmad et al.(57) | 2016 | Rat | 63 | Transient MCAO, filament | Chitosan nanoparticles | Rutin | 0 h | IN | Y | Grip strength, open field |
| Ahmad et al.(58) | 2017 | Rat | 42 | Transient MCAO, filament | Mucoadhesive nanoemulsion | Safranal | 0 h | IN or IV | Y | Grip strength, open field |
| Ahmad et al.(59) | 2018 | Rat | 63 | Transient MCAO, filament | Mucoadhesive nanoemulsion | Quercetin | 21 days pretreatment | IN or IV | Y | Grip strength, open field |
| Bao et al.(60) | 2018 | Rat | NK | Transient MCAO, filament | Ceria nanoparticles | Edaravone | 0 h | IV | Y | N/A |
| Bible et al.(61) | 2009 | Rat | 20 | Transient MCAO, filament | PLGA microparticles | Mouse NPCs | 2 weeks | IC | Y | N/A |
| Chen et al.(62) | 2010 | Rat | 36 | Permanent MCAO, ligation | Fibrin glue | Mouse iPSC derived NPCs | 1 h | IC | Y | Grip strength, rotarod |
| Cook et al.(34) | 2017 | Mouse | NK | Transient, N5-(1-iminoethyl)-L-ornithine/permanent, photothrombotic | Hyaluronic acid hydrogel | BDNF | 7 days | IC | Y | Cylinder test, grid walking |
| Darabi et al.(63) | 2017 | Rat | 66 | Transient MCAO, filament | Fullerenol nanoparticles | N/A | 30 mins before | IP | Y | Neurological score |
| Emerich et al.(64) | 2010 | Rat | 35 | Transient MCAO, filament | Alginate hydrogel | Recombinant VEGF | 0 h | IC | Y | Elevated body swing test, neurological score |
| Fabian et al.(26) | 2018 | Rat | 72 | Transient MCAO, filament | Carbon PEG nanoparticles | N/A | 0 + 2 h | IV | Y | Neurological score |
| Feczko et al.(65) | 2019 | Rat | 27 | Transient MCAO, filament | Theranostic albumin nanocarriers | NGF + U0126 | 3 days | IV | Y | N/A |
| Foroshani et al.(66) | 2018 | Rat | 111 | Transient MCAO, filament | Fullerenol nanoparticles | N/A | 30 mins before + 0 h | IP | Y | Neurological score |
| Fukuta et al.(67) | 2016 | Rat | 28 | Transient MCAO, filament | Liposomes | Fasudil | 0 h | IV | Y | Neurological score |
| Gaudin et al.(68) | 2014 | Mouse | 70 | Transient MCAO, filament/ Permanent MCAO | Squalenoyl adenosine nanoparticles | N/A | 0/2 h | IV | Y | Neurological score |
| George et al.(20) | 2018 | Rat | 60 | Permanent distal MCAO, electrocoagulation | C7 recombinant protein and PEG-peptide hydrogel | VEGF-A and MMP9 | 7 days | IC | Y | Vibrissae-forepaw test |
| George et al.(21) | 2017 | Rat | 60 | Permanent distal MCAO, electrocoagulation | Polypyrrole scaffold | Human NPCs | 7 days | IC | Y | Neurological score, vibrissae forepaw test |
| Ghuman et al.(38) | 2017 | Rat | 22 | Transient MCAO, filament | Hydrogel, ECM | N/A | 14 days | IC | Y | Bilateral asymmetry test, foot fault, amphetamine-induced rotation bias |
| Ghuman et al.(32) | 2018 | Rat | 56 | Transient MCAO, filament | Hydrogel, ECM | N/A | 2 weeks | IC | Y | N/A |
| Guo et al.(69) | 2018 | Mouse | 40 | Transient MCAO, filament | PEG-PCL nanoparticles | Glyburide | 0 + 24 + 48 h | IV | Y | Neurological score |
| Harris et al.(70) | 2016 | Mouse | NK | Transient MCAO, filament | PEG-PGA nanoparticles | BDNF | 3 + 24 h or 6 + 24 h or 12 + 24 h | IV | N | Neurological score, novel object, tail suspension test |
| Hassanzadeh et al.(71) | 2017 | Rat | 30 | Transient global ischaemia, four-vessel occlusion | Nanostructured lipid carriers | Sesamol | 4 days before + 0 h | IV | Y | Neurological score, Morris water maze, step-through passive avoidance test |
| Hayon et al.(25) | 2012 | Rat | 21 | Permanent distal MCAO, electrocoagulation | Platelet microparticles | N/A | 0 h | IC | Y | Neurological score |
| Hazekawa et al.(72) | 2012 | Rat | 88 | Transient MCAO, filament | PLGA microspheres | ONO‐1301 | 1 h or 6 h or 24 h | SC | Y | Neurological score |
| Hettiatatchi et al.(73) | 2019 | Rat | 41 | Transient, endothelin-1 | Methylcellulose hydrogel | Chondroitinase ABC | 7 days | IC | Y | N/A |
| Hu et al.(74) | 2018 | Mouse | NK | Permanent distal MCAO, FeCl3 thrombosis | Iron oxide microrods | tPA | 30 mins | IA | Y | N/A |
| Ishii et al.(75) | 2012 | Rat | 35 | Transient MCAO, filament | PEGylated liposomes | Asialo-erythropoietin | 0 h | IV | Y | Neurological score |
| Jin et al.(76) | 2010 | Rat | NK | Permanent distal MCAO, electrocoagulation | Matrigel | Human NPCs | 3 weeks | IC | Y | Adhesive removal test, cylinder test, Y-maze |
| Jin et al.(77) | 2011 | Rat | 36 | Transient MCAO, filament | Gelatin microspheres | HMGB1 A box | 3 h before or 6 h before or 1 h or 3 h or 6 h | IC | Y | Neurological score, rotarod |
| Jin et al.(78) | 2014 | Rat | 200 | Transient MCAO, filament | Gelatin microspheres | Osteopontin | 24 h before or 1 h or 6 h or 12 h | IC | Y | Neurological score, rotarod |
| Jin et al.(79) | 2017 | Mouse | NK | Transient MCAO, photothrombotic | PEG-PLA micelles | Edaravone | 1, 4, 7 days | IV | Y | Foot fault, neurological score |
| Joachim et al.(80) | 2014 | Rat | NK | Transient MCAO, filament | Gelatin nanoparticles | osteonpontin | 1 h/ 3 h/ 6 h | IN | Y | N/A |
| Klose et al.(81) | 2009 | Rat | 18 | Transient MCAO, filament | PLGA microparticles | Fenofibrate | 24 h before | IC | Y | N/A |
| Lee et al.(82) | 2011 | Rat | 456 | Transient MCAO, filament | Amine-modified single-walled carbon nanotubes | N/A | 1 week before | ICV | Y | Rotarod |
| Liu et al.(83) | 2013a | Rat | 30 | Transient MCAO, filament | Cationic bovine serum albumin-conjugated PEGylated tanshinone IIA nanoparticles | N/A | 0 h | IV | Y | Neurological score |
| Liu et al.(84) | 2013b | Rat | 20 | Transient MCAO, filament | Cationic bovine serum albumin-conjugated PEGylated tanshinone IIA nanoparticles | N/A | 0 h | IV | Y | Neurological score |
| Liu et al.(85) | 2017 | Rat | 45 | Transient MCAO, filament | PEG melanin nanoparticles | N/A | 0 h | ICV | Y | N/A |
| Lv et al.(86) | 2018 | Rat | 49 | Transient MCAO, filament | Boronic ester modified dextran polymer nanoparticles | NR2B9C | 2 h | IV | Y | Neurological score |
| Marushima et al.(87) | 2011 | Rat | 56 | Transient MCAO, filament | Radical-containing  nanoparticles | TEMPO free radicals | 30 mins | IV | Y | Neurological score |
| Matsuse et al.(88) | 2011 | Rat | 33 | Transient MCAO, filament | Collagen sponge | Rat MSC-derived neural progenitors + bFGF | 7 days | IC | Y | Beam balance test, limb placing test, Morris water maze |
| Mdzinarishvili et al.(89) | 2013 | Mouse | NK | Transient MCAO, filament | PLGA-b-PEG nanoparticles | Triiodothyronine | 0 h | IV | Y | N/A |
| Moon et al.(90) | 2012 | Rat | 50 | Transient MCAO, filament | Carbon nanotubes | Mouse NPCs | 24 h | IC | Y | Rotarod, treadmill test, vibrissae forelimb test |
| Moshayedi et al.(91) | 2016 | Mouse | NK | Permanent, photothrombotic | Hyaluronic acid hydrogel | Human iPSC-derived NPCs | 7 days | IC | Y | N/A |
| Nagai et al.(92) | 2015 | Mouse | NK | Transient MCAO, filament | Cilostazol nanoparticles | N/A | 3 h | IV | Y | Neurological score |
| Nih et al.(93) | 2018 | Mouse | 72 | Permanent distal MCAO, photothrombotic | Hyaluronic acid hydrogel | Heparin nanoparticle-bound VEGF | 5 days | IC | Y | Cylinder test, grid walking, pasta test |
| Parent et al.(94) | 2015 | Rat | 41 | Transient MCAO, thromboembolic | PLGA microparticles | S-Nitrosoglutathione | 2 h | SC | Y | Neurological score |
| Payne et al.(33) | 2019 | Rat | 75 | Transient, endothelin-1 | Hyaluronan and methylcellulose hydrogel | Human iPSC-derived NEPs | 7 days | IC | Y | Montoya staircase, tapered beam test |
| Quittet et al.(23) | 2015 | Rat | 133 | Transient MCAO, filament | PLGA microspheres | Rat MSCs/rat MSCs + VEGF | 24 h | IC | Y | Adhesive removal test, beam walking, corner test, limb placing test, neurological score |
| Ramos-Cabrer et al.(95) | 2011 | Rat | 19 | Permanent MCAO, ligation | Liposomes | Citicoline | 30 min, 6, 12, 18, 24 + 30 h | IV or IP | Y | N/A |
| Ravina et al.(96) | 2018 | Rat | 96 | Permanent distal MCAO, electrocoagulation | Hyaluronic acid hydrogel | BDNF | 8 days | IC | Y | Adhesive removal test, neurological score |
| Reddy et al.(97) | 2009 | Rat | 27 | Transient MCAO, filament | PLGA nanoparticles | Superoxide dismutase | 0 h | IV | Y | Neurological score |
| Sanchez-Rojas et al.(39) | 2018 | Mouse | 24 | Permanent MCAO, FeCl3 thrombosis | Hyaluronic acid scaffold | Human adipose stem cells | 7 days | IC | Y | N/A |
| Saraiva et al.(22) | 2018 | Mouse | 73 | Transient MCAO, photothrombotic | PLGA nanoparticles | miR-124 | after recovery anaesthetic | IV | Y | Grid walking, rotarod |
| Shimbo et al.(24) | 2017 | Rat | 116 | Transient MCAO, filament | PEG liposomes | Haemoglobin | 0 h | IA | Y | Neurological score |
| Takamiya et al.(98) | 2012 | mice | 86 | Transient MCAO, filament | Platinum nanoparticles | tPA | 0 h | IV | Y | Neurological score, rotarod |
| Tan et al.(99) | 2018 | Rat | 40 | Transient MCAO, clamps | Luteolin micelles | N/A | 0 h | IV | Y | N/A |
| Vani et al.(100) | 2016 | Rat | 48 | Transient MCAO, filament | Fullerene nanoparticles | N/A | 30 mins before or 0 h | IP | Y | Neurological score |
| Wang et al.(101) | 2012 | Mouse | 54 | Transient, endothelin-1 | Hyaluronan/methyl cellulose hydrogel | Erythropoietin | 4 or 11 days | IC | Y | N/A |
| Wang et al.(102) | 2015 | Rat | 60 | Transient MCAO, filament | Soya lecithin/cholesterol/PEG-DSPE/T7-PEG-DSPE liposomes | ZL006 | 0h | IV | Y | Neurological score |
| Wang et al.(103) | 2018 | Mouse | 25 | Transient MCAO, filament | PEG-PLA nanoparticles | siRNA complement component C3 | 0 h | IV | Y | Neurological score |
| Yan et al.(104) | 2015 | Rat | 21 | Transient MCAO, filament | Chitosan-collagen scaffold | Rat MSCs | 24 h | IC | N | Neurological score |
| Yang et al.(105) | 2019 | Mouse and rat | NK | Transient MCAO, filament | NOE and soybean phosphatidylcholine nanoparticles | N/A | 0 h then daily for 14 days | IV | Y | Beam walking, grip strength, Morris water maze, neurological score |
| Yu et al.(106) | 2010 | Rat | 100 | Transient MCAO, filament | Collagen sponge | Rat NSCs | 24 h | IC | N | Neurological score |
| Yun et al.(107) | 2013 | Mouse | NK | Transient MCAO, filament | n-BCA nanoparticles/PLGA nanoparticles/liposomes | Superoxide dismutase | 0 h | IA | Y | Neurological score |
| Zhang et al.(108) | 2017 | Rat | NK | Transient MCAO, filament | Plasma scaffold | Rat MSCs | 3 weeks | IC | Y | Elevated body swing test, cylinder test, ladder walking, limb placing test, neurological score |
| Zhang et al.(109) | 2018 | Rat | 85 | Transient MCAO, filament | Ceria nanoparticles | LXW7 | 1 h | IV | Y | Neurological score |
| Zhao et al.(110) | 2013 | Rat | 48 | Transient MCAO, filament | n-BCA nanoparticles | Puerarin | 0 h | IV | Y | Neurological score |

Abbreviations: BDNF, brain-derived neurotrophic factor; bFGF, basic fibroblastic growth factor; DSPE, 1,2-distearoyl-sn-glycero-3-phosphoethanolamine; ECM, Extracellular matrix; HMGB1, high mobility group box 1 protein; IA, intra-arterial; IC, intracranial; ICV, intracerebroventricular; IN, intranasal; IP, intraperitoneal; iPSCs, induced pluripotent stem cells; IV, intravenous; n-BCA, n-butyl cyanoacrylate; MCAO, middle cerebral artery occlusion; miRNA, micro-ribonucleic acid; MMP, matrix metalloproteinase; MSCs, bone marrow-derived mesenchymal stem cells; NEPs, neuroepithelial progenitor cells; NGF, nerve growth factor; NK, Not known; NMP, N-Methyl-2-Pyrrolidone; NOE, N-oleoylethanolamine; NPCs, neural progenitor cells; PCL, polycaprolactone; PEG, poly(ethylene) glycol; PGA, poly-L-glutamic acid, PLA, Poly(lactic) acid; PLGA, poly(lactic-co-glycolic) acid; SC, subcutaneous; siRNA, small interfering ribonucleic acid; TEMPO, 2,2,6,6-tetramethylpiperidine-1-oxyl; tPA, tissue plasminogen activator; VEGF, vascular endothelial growth factor.
